# Gut microbiome development in early childhood is affected by day care attendance

**DOI:** 10.1038/s41522-021-00265-w

**Published:** 2022-01-11

**Authors:** Amnon Amir, Ortal Erez-Granat, Tzipi Braun, Katya Sosnovski, Rotem Hadar, Marina BenShoshan, Sophia Heiman, Haya Abbas-Egbariya, Efrat Glick Saar, Gilat Efroni, Yael Haberman

**Affiliations:** 1 Sheba Medical Center, Tel-HaShomer, affiliated with the Tel-Aviv University, Tel-Aviv, Israel; 2 Cincinnati Children’s Hospital Medical Center and the University of Cincinnati College of Medicine, Cincinnati, OH USA

**Keywords:** Microbiome, Metagenomics

## Abstract

The human gut microbiome develops during the first years of life, followed by a relatively stable adult microbiome. Day care attendance is a drastic change that exposes children to a large group of peers in a diverse environment for prolonged periods, at this critical time of microbial development, and therefore has the potential to affect microbial composition. We characterize the effect of day care on the gut microbial development throughout a single school year in 61 children from 4 different day care facilities, and in additional 24 age-matched home care children (*n* = 268 samples, median age of entering the study was 12 months). We show that day care attendance is a significant and impactful factor in shaping the microbial composition of the growing child, the specific daycare facility and class influence the gut microbiome, and each child becomes more similar to others in their day care. Furthermore, in comparison to home care children, day care children have a different gut microbial composition, with enrichment of taxa more frequently observed in older populations. Our results provide evidence that daycare may be an external factor that contributes to gut microbiome maturation and make-up in early childhood.

## Introduction

Early childhood is an important period where different interventions can affect behavioral and biological factors, supporting a child’s growth. Early childhood is also a pivotal time for the development of the gut microbial composition and the host immune system. During this time, the gut microbial composition displays the highest intra- and inter-individual variability^1–3^. Microbial maturation likely facilitates host immune maturation^4,5^. In germ-free animals, in the absence of the microbiota, there is a deleterious effect on the immune system^6^ and on brain development^7^. Behavioral and cognitive developments take place parallel to microbial maturation in the first years of life. Previous studies have demonstrated a short-term beneficial impact of preschool participation on early cognitive skills^8^, and some long-term effects on health, educational attainment, and earnings^9–11^. A recent study has also connected the taxonomic and functional composition of the gut microbiome with behavior during early school-aged children’s development^12^.

Previous studies demonstrate that the gut microbiome composition is established within the first 3 years of life, where it is predominantly shaped by environmental factors, and not by genetics^13,14^. Studies have focused on the first year of life, showing that mode of delivery and breastfeeding impact the microbial composition. Children in this age group are mostly exposed to their family, but, once they grow and start attending day care, they become exposed to additional environmental factors as well as new children and adult caregivers, all of which may have a significant effect on their microbial composition. Few previous studies looked at the microbiota in relation to daycare attendance. One study included 9 infants, and tested the effect of feeding on the infant microbiome and also showed that day care attending infants harbor higher microbial diversity compared to non-daycare attending infants^15^. A second study compared 98 home vs. day care infants at the age of 3 months, but failed to identify differences after a 4 week follow-up^16^. A third study collected stool samples over the course of a year aiming to characterize stability, inter- and intra-individual variability and resilience to antibiotic use and illness, but did not compare those results to a home care group^17^. In our current work, we followed a cohort of children (*n* = 61) attending four different day care facilities for their first time, aiming to evaluate the effect of day care attendance on the microbial composition throughout a day care year (up to 5 samples per child). We compared those to an additional cohort of 24 age-matched children in home care. Using this dataset, we show that day care attendance is a significant factor impacting microbial composition and maturation.

## Results

### Characteristics of cohort

**Fig. 1:**
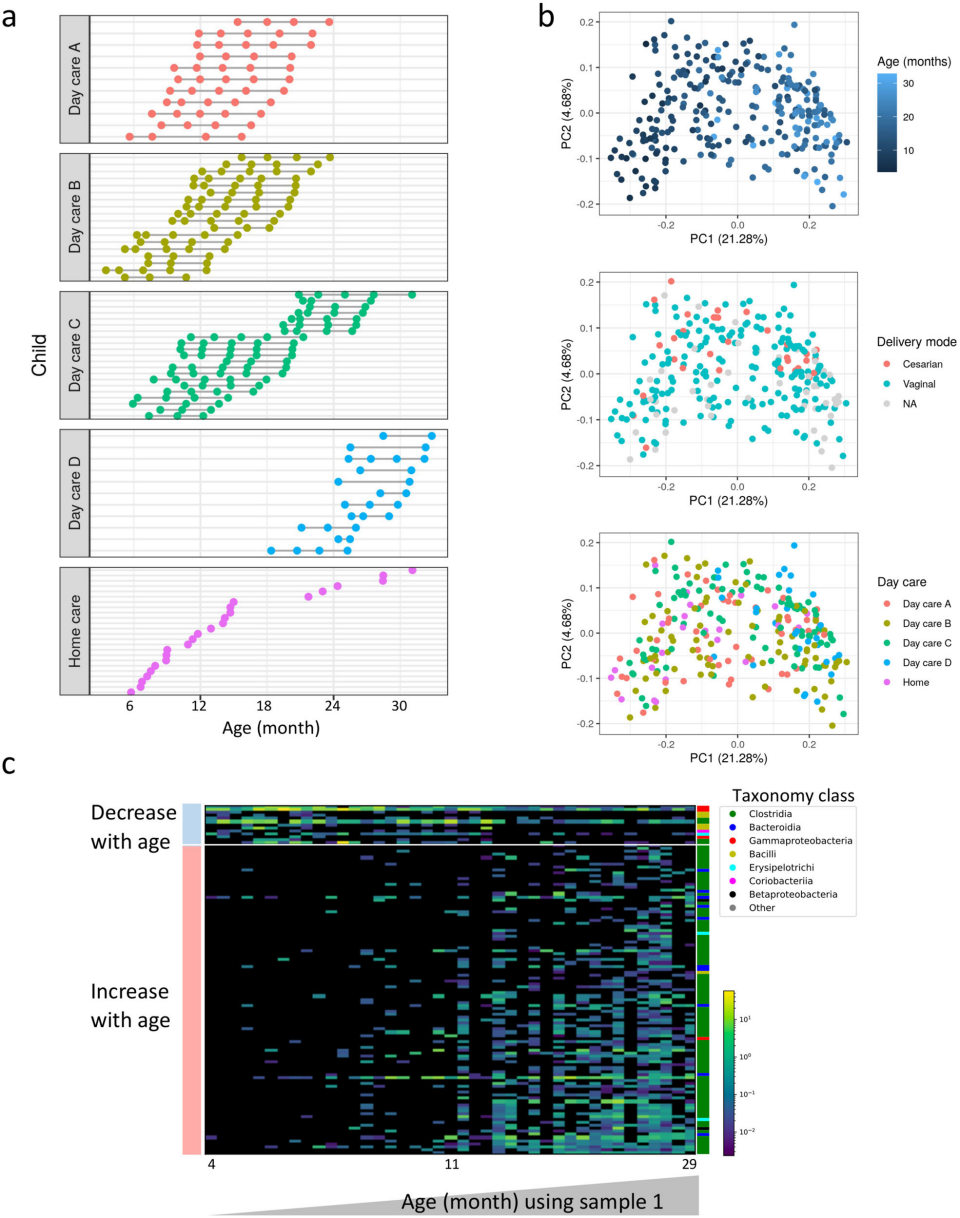
Longitudinal cohort of 61 children starting day care, and additional age-match home care children. **a** Each row corresponds to an enrolled child with longitudinal fecal sampling. Stool samples were collected within the first 2 weeks after each child started persistent day care attendance (sample or time point 1), and after 2 months (time point 2), 4 (time point 3), 7 (time point 4), and 10 months (time point 5) during the same school year. **b** Unweighted UniFrac PCoA plots colored by age, mode of delivery, and day care or home care of the 268 samples included in our study. **c** A heatmap representing 116 amplicon sequence variants (ASVs) that showed significant correlation with age in day care children using the first sample obtained within the first 2 weeks after each child started persistent day care attendance (Spearman correlation *r* > 0.3, dsFDR < 0.1). Each row represents a different ASV and each column a different sample. Samples are ordered by age, ASVs ordered by the effect size, color bar on the right indicates ASVs taxonomy class. Source data are provided as a Source Data file.

We prospectively collected stool samples and metadata from 61 children in 4 different day care facilities (day care A, B, C, D) from the same geographical region (Fig. 1a and Table 1). In Day care B and C there were 2 classes within each facility, with some of the caregivers working in both classes. Samples and metadata were collected within the first 2 weeks after each child started consistent day care attendance (sample/time point 1), and after 2, 4, 7, and 10 months (time points 2–5) during the course of the year. In addition, we collected samples from 24 age-matched home care children (Table 1). Home care children did not attend day care before or during the study sampling. All four day care facilities had internal variable home-made cooking (including dairy products, fruits, vegetables, chicken, pasta, and rice). Median age of entering the study was 12 months and the groups included 49%/58% males in daycare/homecare respectively. Median numbers of subjects in the household were 4 and most were delivered vaginally (86% in the day care and 83% in the home care). The minority of the children were never breast fed (11% in those attending day care and 26% at home care), there were 63% in the day care group and 53% of the home care group that were still breast fed, and all children were already taking complementary solids. Altogether, day care and home groups did not vary significantly in demographics and characteristics features tested (Table 1).

**Table 1:** Cohort demographics and characteristics.

	*N*	Day care^b^	*N*	Home care
		*n* = 61		*n* = 24
Age month Median (IQR)^a^	61	11.1 (9, 19.6)	24	12.6 (8.7 16.7)
Male (*n*, %)	61	30 (49%)	24	14 (58%)
Number of persons in household Median (IQR)	49	4 (3,4)	24	4 (3,5)
Breast feeding				
Never breast fed	38	4 (11%)	23	6 (26%)
Still some breastfeeding during sampling^1^	38	24 (63%)	15	8 (53%)
Mode of delivery				
Vaginal delivery	49	42 (86%)	24	20 (83%)
Cesarian delivery	49	7 (14%)	24	4 (16%)
Antibiotic within 3 days after delivery	34	1 (3%)	23	3 (13%)
Antibiotic during delivery	34	1 (3%)	23	4 (17%)

^a^Age and breastfeeding when entering the study.

^b^Normally distributed continuous variable (age) was compared using two-sample *t*-test. Non-continuous variables were compared using *χ*2 test between groups. Group were not significantly different (*p* > 0.05) in the variable indicated.

### Age is a dominant factor in gut microbial maturation

We performed V4 16S rRNA amplicon sequencing, constructed amplicon sequence variants (ASVs) profiles for all 268 samples, and characterized ASVs relative abundance patterns. An unweighted UniFrac based Principal Coordinates Analysis (PCoA) was performed to visually explore the similarity and variation between samples’ microbial composition (Fig. 1b). As previously shown^2^, age is a dominant confounder of the microbial composition not only in the first, but also in the second and third years of life, as older kids cluster more toward the right of the PCoA plot (Fig. 1b), with PC1 values highly correlated with age [Spearman’s rank correlation rho using one random sample per child = 0.72, *p*-value < 1.5e-14, and repeating the analyses using 100 random iterations resulted in rho values ranging from 0.64 to 0.78 (mean *ρ* = 0.72, sd = 0.027) and *p* values ranging from 9.09e-19 to 3.40e-11 (mean *p*-value = 6.75e-13, sd = 3.73e-12)]. PCoA results colored by mode of delivery or by day care facility are shown in Fig. 1b. ASVs that are significantly positively or negatively correlated with age in day care children using only the first sample per child are shown in Fig. 1c (Spearman *r* > 0.3, dsFDR<0.1, Supplementary Data Source). These results further highlighted the known effect of age on the gut microbial maturation, and emphasize the need for age adjustment, as used throughout in our downstream analyses.

### Day care facility and class are significant contributors to gut microbial composition

**Fig. 2:**
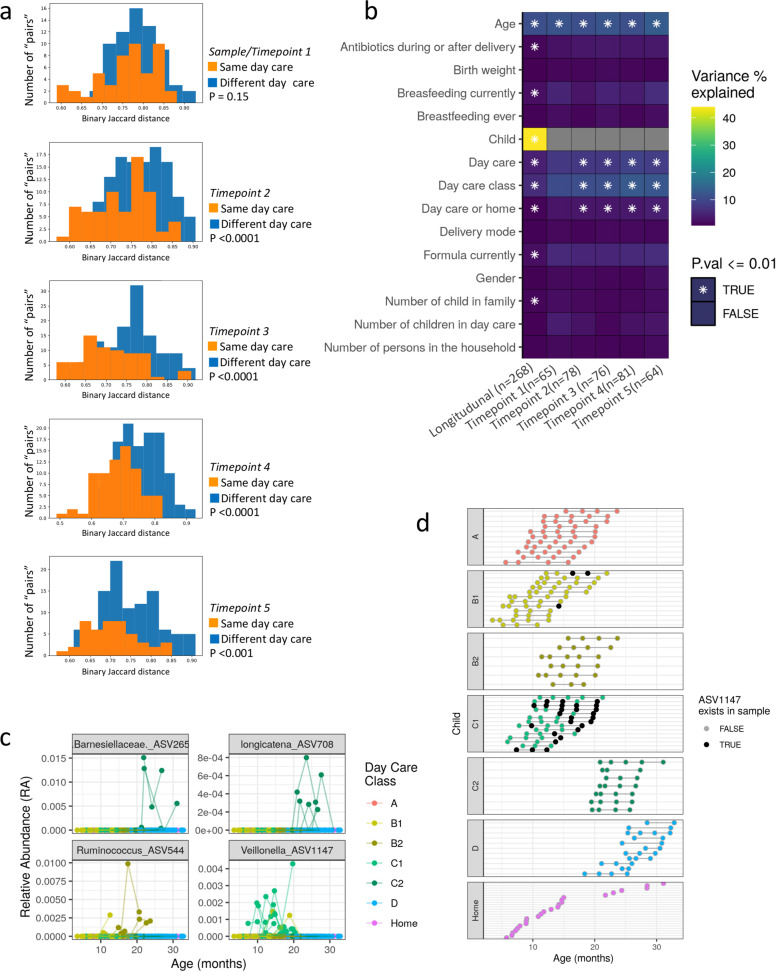
Specific day care is a significant contributor to gut microbial composition in early childhood. **a** Binary Jaccard distance was calculated between pairs of age-matched children (born within up to 1 month apart). Distances are shown for age-matched children pairs participating in the same day care (orange) or a different day care facility (blue), showing that from the second sampling onward, age-matched pairs from the same day care share more of their microbiome in comparison to pairs from different day care facilities. *P*-values for differences between same and different day care pairs were calculated using a two-sided Mann–Whitney test. **b** PERMANOVA shows that inter-individual variation explains most (44%) of the variation when using longitudinal sampling, followed by age (11%), day care class (6%), and day care facility (4%) (left most column). The effect of day care facility and class is further noted when examining each sampling point separately, where the variation explained by day care class increases to 12% in the fourth and fifth sampling point. Stars show statistical significance (*P ≤ 0.01). Variance is estimated for each feature independently, while accounting for age, gender, and subject when needed (see Methods section). Total *n* is shown in brackets. **c** Age against relative abundance of 4 ASVs significantly associated with day care class in the maaslin2 analysis (see Methods section). Barnesiellaceae appeared in 4 of the 7 children in day care C class 2, and in none of the other children. Longicatena also appeared in 4 of the 7 children in day care C class 2, and in none of the other children (not the same 4 children as Barnesiellaceae). Veillonella appeared in 12 of the 14 children in day care C class 1, and in additional 2 children from day care B class 1. Lastly, Ruminococcus appeared in 4 of the 12 children in day care B class 1, and in 4 of the 6 children in day care B class 2, and in none of the other day cares. **d** A cohort figure showing all children from specific day care classes over age, similar to Fig. 1a. Samples positive to Veillonella (ASV1147, Supplementary Data Source) are marked in a black circle. Source data are provided as a Source Data file.

In order to evaluate the contribution of day care facility to the gut microbial community, a naïve approach would be to compare beta diversity distances between participants from same vs. different day care facilities. However, since there are differences in the age distributions between the different day care facilities tested, this can lead to confounding effects due to age-related microbiome changes. To overcome the confounding effect of age, we limited the beta diversity distance analysis only to age-matched pairs of children (defined as those within a one month different in age). Figure 2a shows the Binary Jaccard distance distribution for age-matched pairs of children participating in the same (orange) or different (blue) day care facilities [as described in methods and using equation (1)]. This comparison was performed separately for each of the sampling time points, and therefore contains a single sample for each participating child. Age-matched pairs from the same day care were significantly more similar (showed significantly lower distance) in comparison to age-matched pairs from two different day care facilities. This effect was noted only from the second sampling onward to the third, fourth, and fifth sampling (Mann–Whitney test *p* < 0.001). No significant difference was noted in the first sampling point, which was taken just as the child entered day care. These results emphasize that from the second sampling onward children from the same day care become more similar in their microbial composition, implying that the specific day care that the child participated in contributed to the overall microbial composition of that specific child. The Jaccard metric is based on presence/absence and does not take into account the relative abundances of the bacteria, and we opted to use it specifically to look for the appearance/disappearance of bacterial ASVs following day care attendance. However, a similar behavior is observed when using Bray–Curtis distances which shows that results are robust, independent of the distance metric chosen (Supplementary Fig. 1), and when using 10 random 4000 reads/sample rarefication (Supplementary Fig. 2).

To quantify the contribution of different factors affecting the gut microbial composition, we used a PERMANOVA test (Fig. 2b and Supplementary Data Source). PERMANOVA was applied in parallel on all longitudinal samples while controlling age, gender and subject (see Methods section), and also by analyzing each sampling point separately (including only one sample per child) to more rigorously control for subject’s contribution, after controlling for age and gender. When considering all samples, intra-personal composition explained the greatest amount of variance (44%), followed by age (11%), and day care class and day care facility (6% and 4%). Other factors showing modest but significant (*p* < 0.01) contributions were gender, time form entering day care, attending day care vs. being home cared, whether the mother or child received antibiotic during and within 3 days of delivery, and breastfeeding together with formula and/or solids. When considering each sampling time point separately and hence only one samples per subject, day care facility, class, and age remained the major significant contributors associated with the microbiome profiles, unlike breastfeeding (ever or currently) and mode of delivery that were not significant. Interestingly, in sample 4 and 5 both day care class and day care facility contribution were similar to that of age (9–13%), and being in home care or day care contributed 3–4% of the microbial variance. These results indicate that the specific day care class is an impactful factor in child microbial compositional makeup. This was consistent also when using 50 random 4000 reads/sample rarefication (Supplementary Fig. 3). To capture day care class-specific microbial ASVs and taxonomy we used the Maaslin2 (Multivariate Association with Linear Models) pipeline after controlling for subject, gender, and age. Overall, 16 unique ASVs were significantly associated with day care class, with *q*-value < 0.05 (ASVs numbers and sequences are indicated in Supplementary Data Source). Example for the distributions of these specific ASVs taxa are shown (Fig. 2c, d). Those include *Veillonella* (ASV1147) which is present in day care C class 1 (Day care C1, Maaslin2 *q* = 0.006), *Dorea longicatena* (ASV708) in Day care C class 2 (day care C2, Maaslin2 *q* = 0.004), and *Ruminococcus* (ASV544) in day care B class 2 (day care B2, Maaslin2 *q* = 0.015).

### Day care children have a different microbial composition than home care children with enrichment of taxa more frequently seen in older populations

To define a more general contribution of attending day care and to capture the differences in microbial composition between day care and home care children, we looked at the community level (alpha-diversity) and at specific ASVs taxa. Supplementary Fig. 4a shows the intra-personal diversity (number of ASVs, as a measure of alpha-diversity) plotted as a function of age, and colored by day care and sampling time point (blue) or home care (red). Overall alpha-diversity increased with age (Pearson correlation *r* = 0.65, *p*-value = 2.2e-30 for day care children, *r* = 0.67 *p*-value = 0.0003 for home care children) in all children as was previously shown in other cohorts^1–3^. Alpha-diversity (the number of species) in the two groups displayed a similar positive dependence on age with slope of 3.4 for day care and 2.1 for home care using linear regression for the number of species as a function of age for the two groups (*p*-value = 0.07 for rejecting the null hypothesis of similar slopes for the two groups using a non-parametric single-sided test with 1000 random permutation of day care or home care labels, Supplementary Fig. 4). Home care children tended to have a lower mean diversity across all age groups when including only one sample per child in each age group (Supplementary Fig. 4b, Mann–Whitney *p*-values 0.2, 0.06, 0.04, 0.04 for the 5–9, 10–14, 20–24, and 25–29 months age groups respectively). Acknowledging the limitation in the numbers of children in the home care group after subgrouping, there did not seem to be systematic differences in delivery mode, number of household members, and breastfeeding between home care and day care children in all subgroups tested beside lower rate of breastfeeding in the 25–29 months home care children; home care children in this age group (*n* = 3) were never breast fed vs. 86% of the days care children that were breastfed.

**Fig. 3:**
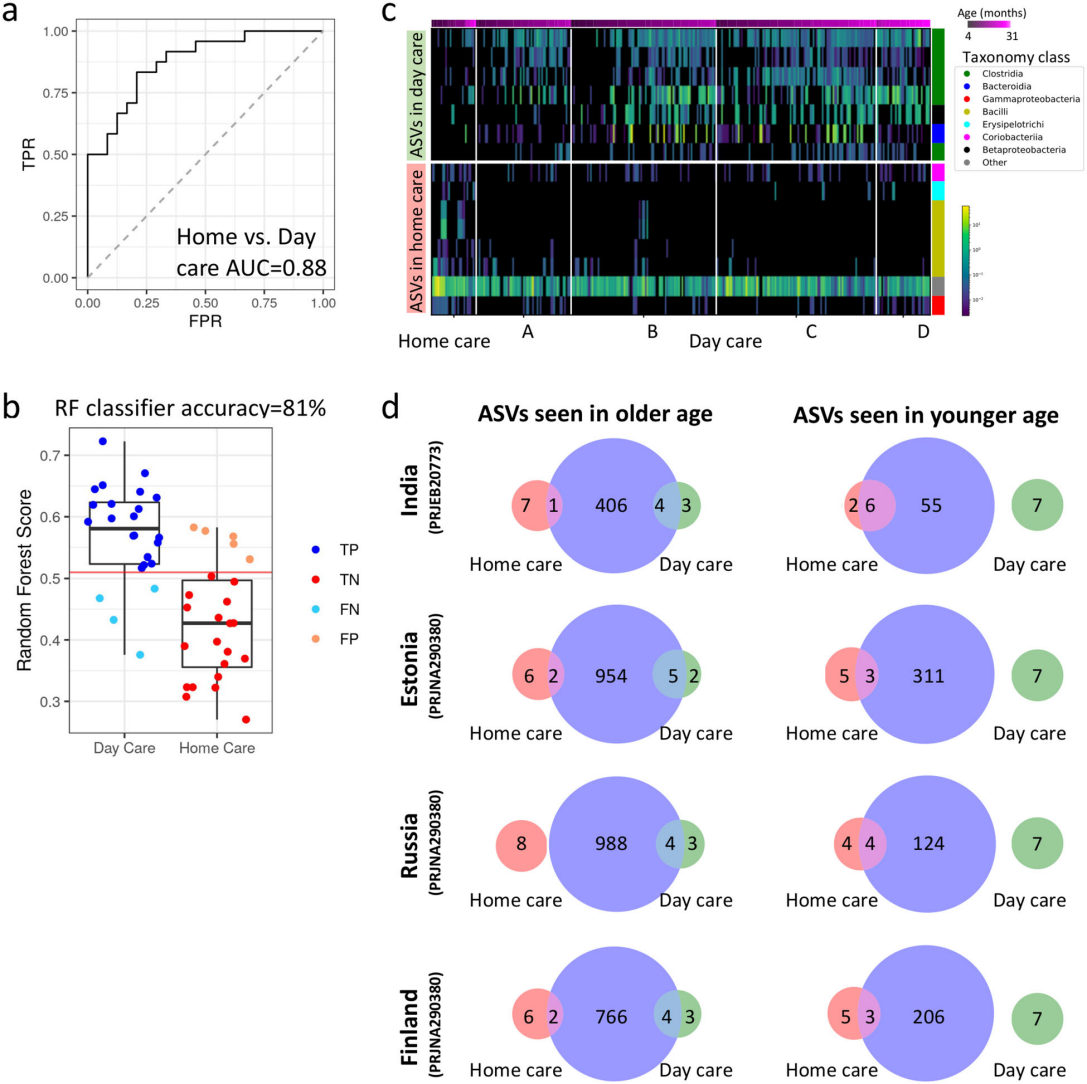
Day care children show a distinct and more mature microbial composition in comparison to age-matched home care children. **a** ROC curve of random forest result, differentiating between 24 home care and 24 age-matched day care samples, with an AUC of 0.88. **b** Random forest out of bag (OOB) score for day care and home care samples with a Youden point threshold of 0.51 (see Methods). True positive (TP), true negative (TN), false positive (FP) and false negative (FN) classification results are colored. **c** A heatmap showing ASVs with significant differential abundance between home care and age-matched day care children samples (paired rank-mean test with dsFDR < 0.1 multiple hypothesis correction, see methods). Each row represents a different ASV (7 ASVs more abundant in day care and 8 more abundant in home care) and each column a different sample. Samples within each day care facility are ordered by age, ASVs are ordered by the effect size, and color bar on the right indicates ASVs taxonomy class. **d** Venn diagram showing overlap between the 7 day care and 8 home care associated ASVs from panel **c** (green and red circles respectively) with age younger/older age associated ASVs (blue circle in right and left columns respectively) identified from other cohorts [PRJNA290380^20^], PRJEB20773^19^] - see Methods section for additional details), emphasizing significant larger overlap of home care enriched ASVs with younger subjects (*χ*^2^ *p* < 0.05) in contrast to day care enriched ASVs that show a more substantial overlap with older subjects. Source data are provided as a Source Data file.

To further evaluate the microbiome ability to discriminate between children at home care and in day care, we used a supervised Random Forests (RF) classifier using a group of age-matched children (one sample from each child) including 24 children from home care (median age 12.4 months IQR 8.7, 16.7) and 24 children from day care (median age 11.4, IQR 8.9, 16.9). In this analysis, we manually selected samples best matching in age (up to one month apart), gender, mode of delivery, breastfeeding and number of persons in the household (Supplementary Table 1), in order to equalize the group sizes used for the random forest classifier and control for potential confounders. A Receiver operating characteristic (ROC) area under the curve (AUC) of 0.88 was obtained (Fig. 3a, b, and the top ASVs taxa used for the classification are shown in Supplementary Fig. 5 and in Supplementary Data Source). To further ensure that this is not the result of a bias in the age-matched sample selection, we performed another random forest analysis, using the 24 home care children and 100 random subsets of 24 age-matched daycare children out of the available age-matched samples. This analysis resulted in AUC values ranging from 0.69 to 0.92, with a median of 0.81. Together, these results indicate that day care children have different microbial composition in comparison to home care children.

Differentially abundant ASVs (between day care and home care) were identified using a non-parametric rank mean test as implemented in Calour^18^ with dsFDR multiple hypothesis correction (FDR < 0.1). To account for age, and to avoid multiple sampling from the same participant, we again used aged-match pairs. Each home care child was matched with day care children that are up to 1 month apart in age, and only one sample per child was included. This resulted in 60 samples from children in day care, which were age matched to the 24 home care samples (median age of 12 and 12.4 months for day care and home care respectively, Supplementary Fig. 6). Differentially abundant ASVs were then detected using a permutation-based non-parametric rank-mean test paired on the matched group (i.e., pairing on the home care participant and all age-matched day care participants), with FDR controlled to 0.1. There were 8 resulting ASVs that were significantly higher in the home care group and 7 that were significantly higher in the day care group (Fig. 3c, taxonomy and specific sequences of those ASVs are in Supplementary Data Source). Increased abundance in home care children were noted for taxa from Bifidobacteriaceae (*q* = 0.04) families of the Actinobacteria phyla, Lactobacillaceae (*q* = 0.05) and Staphylococcaceae (*q* = 0.05) of the Firmicutes phyla, and Pasteurellaceae (*q* = 0.04) of the Proteobacteria family. Increased abundance in day care children were noted for Prevotellaceae family and Prevotella genus of the Bacteroidetes phyla (*q* = 0.04), and Lachnospiraceae (*q* = 0.05) and Ruminococcaceae (*q* = 0.04) of the Firmicutes phyla.

The presence of Bifidobacteriaceae and Lactobacillaceae were previously more related with younger children and infants, whereas Prevotellaceae is more seen in older children and adults^19^, we therefore tested if these 15 ASVs that differ between day care and home care were also linked with age-dependent maturation in other studies. We used two publicly available datasets with V4 16S amplicon sequencing, spanning four different countries from different geographic regions that included children in similar and older age groups (PRJEB20773^20^) and children and adults (PRJNA290380^21^). Each of these studies was processed using the same pipeline used in the current study, to enable direct ASV comparison. Age-related ASVs in each of these studies were identified by testing for significantly positively or negatively correlated with age in each of the three countries (Estonia and Finland with higher and Russia with lower autoimmune prevalence) in PRJNA290380^21^ or differentially abundant in adults vs. children in India in PRJEB20773^20^. We then tested how many of the ASVs differentiating between home care and day care in our current study were also observed as age-related ASVs in each of these comparisons across 4 different countries. Figure 3d shows the overlap between the ASVs observed in our study as higher in day care (green circles) or home care (red circles), and ASVs associated with old or young age in these additional studies (blue circles in left and right columns respectively). More than half of the ASVs observed as higher in day care children in our current study were associated with older age in the additional studies tested (left column), whereas none were associated with younger age (right column) (*χ*^2^ *p* < 0.05). Conversely, ASVs observed as higher in home care children in our current study showed a larger overlap with ASVs associated with younger age (right column) compared to ASVs associated with older age or adults (left column). We therefore concluded that day care children not only have distinct microbial composition but those bacterial ASVs seen in day care children were also reported in older children and adults, rather than in younger children.

## Discussion

**Fig. 4:**
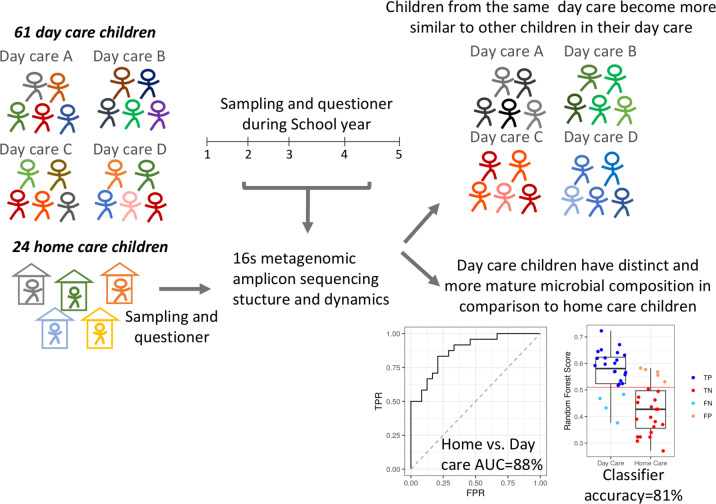
Cartoon highlighting the results implicating that day care environment is a significant factor impacting microbial dynamics in the second and third year of life. The specific day care facility is shaping the growing child’s microbial composition, with each child becoming distinct and more similar to his classmates. Furthermore, when comparing to home care children, day care children have distinct and more mature microbial composition with enrichment of taxa more frequently seen in older populations.

The longitudinal nature of our cohort allowed us to characterize the dynamics of the microbial composition in early childhood, taking into account day care as a small ecosystem. Unlike studies that focused on the first year of the child’s life and indicated strong contribution for mode of delivery and breastfeeding, we focus and highlight the dynamics taking place in the second and third year of life. In this period, we show a relative minor contribution for mode and delivery and breastfeeding on the microbial variation, and a much stronger contribution of specific day care and class on the microbial variation. We further show that children attending day care have a distinct microbial composition, with enrichment of taxa more frequently seen in older populations, compared to that seen in age-matched children that have not yet started day care, and those variation can be correctly classified using the RF machine learning classifier (Fig. 4 cartoon highlights those findings).

Three published studies have also examined the effect of day care attendance on the child microbiome to some extent^15–17^. The first^15^, primarily looked at the effect of diet in nine children, while indicating also an increase in alpha diversity in the samples following day care attendance. However, this study lacked controls for age-matched non-day care participants, and therefore cannot differentiate between the effect of day care attendance and the effect of aging, which is also linked to increased alpha diversity. Here, we augmented those results comparing the participants to age-matched children from other day care facilities and home schooling. We further show that specific ASVs bacteria are detected in a specific day care class, and are hardly detected in the other day cares or classes (Fig. 2c, d). Additionally, by comparing to other infant studies, we show that the bacteria associated with day care attendance are associated with older age. The second study^16^, focused on children who entered day care at 3 months of age and followed them for only 4 weeks. This study failed to show a significant contribution of day care attendance or a significant difference from home care children. Differences between the experiment design of this study and our current study include the younger age of the children (3 months vs. median of 12 month), the relative short period of follow-up (4 weeks vs. 10 months), and the relative limited period the children attended day care [about 2 days a week, as opposed to a full week (6 days a week)]. As a result of these differences in the experimental design, the different conclusions reached may be due to a faster microbial population change in infancy, leading to a reduced general impact of daycare attendance compared to other environmental factors such as breastfeeding and close contact with the mother. Additionally, it is possible that older children in daycare move more independently, touch more objects, and are in closer contact with other children than young infants in daycare. The third study^17^, had a different design, as it looked at inter- and intra-individual variability and resilience to antibiotic and illness in the microbiota of daycare children aged 1–6 years, without comparison to home care or other day care groups. We therefore believe that while the idea of the effect of day care attendance on the microbiome has been studied before, our study adds a significant and important contribution in identifying early influences on the gut microbial system in humans. Limitations in our study include the relative small number of participants, lack of data regarding additional factors such as household pets, more detailed diet exposures, children’s health status, household characteristics (e.g. parenting stress, quality of care), which may also contribute to gut microbial composition. Additionally, the current study design did not enable testing the minimum number of hours needed to significantly impact the microbiota, the first sample was obtained during the first days of day care rather than prior to day care initiation, and there could be other unmeasured factors influencing whether parents choose homecare vs daycare and that those may influence the child’s gut microbiome. Moreover, early life antibiotics have been shown to effect microbial composition at a later point in time^19,22^. We noted a marginal trend toward higher antibiotics use during or after delivery in the home care group. While PERMANOVA analyses show significant but limited contribution of antibiotics use around birth (1.3%) vs. the 6% contribution of the day care class to the microbial variation, it is possible that antibiotics also contributes to some of the effect seen between day care and home care. Future studies with larger cohort sizes may be able to more robustly address those and additional factors.

Day care participation exposes children to a large group of peers for a prolonged period of time during the day (8 h a day, 5–6 days a week on average). The fact that children from the same day care share more of their microbiota, and the strong effect of a specific day care facility and class on the microbial community emphasize the importance of such environmental intervention on the growing child microbial composition in early childhood. Interestingly, the frequency of day care attendance during early childhood^23^ and population mixing of children^24^ were inversely associated with childhood diabetes, and increasing the number of children in the day care setting was significantly associated with increasing protection from diabetes. These findings suggest that early exposure may play a role in the development of immune-regulatory mechanisms which protect against diabetes. Follow-up longitudinal studies are warranted to examine if and how specific patterns of the gut microbial maturation and day care attendance in healthy children are linked with future health and disease states, as well as immunologic and allergic outcomes^25^.

In this context, the “hygiene hypothesis” was built on the observation that young siblings in large families were less likely to have atopic diseases than older siblings or children from small families^26,27^. The interpretation for this observation was that those kids suffered from more infections in early childhood, interpretation that was later disputed when large cohorts have found no association between the number of viral infections and allergic disease^28^. On the other hand, growing up in a farm and with pets reduces the likelihood of developing asthma^29^, allergies, and inflammatory bowel disease (IBD)^30,31^. It is possible that growing up in a more diverse microbial ecosystem during early childhood helps train the immune system not to overreact to triggers. This was recently shown to be the case, where farm-like indoor microbiota in non-farm homes protected children from asthma development^32^. Day care in that sense is linked to both increase risk for infections^33^ but also based on our study to a more mature and diverse microbial ecosystem, thereby potentially contributing additional explanation to the “hygiene hypothesis”. Interestingly, Haemophilus genera from Pasteurellaceae family have been associated with an increased risk for asthma, when abundant in home dust or colonizing the airways early in life^34^, and it was also more abundant in the gut of home care children in comparison to day care children.

In summary, the gut microbiome is shaped predominantly by environmental factors, while genetics explain <10% of the variation^13,14^, and it plays a crucial role in immune development and function^4,5^, as well as in host metabolic state^35^. The First 3 years of life (early childhood) display the highest intra- and inter-individual gut microbiome variability^1–3^, and it is therefore thought to be the most influential time for the gut microbiome maturation. We show that day care attendance is impactful and significant in shaping the microbial composition in early childhood. The specific day care facility and class influence the gut microbiome, and each child becomes more similar to others in their day care. Furthermore, day care children have a different gut microbial composition in comparison to home care children, with enrichment of taxa more frequently observed in older populations. While the idea of the effect of day care attendance on the microbiome has been studied before, our study adds a significant and important contribution in identifying early influences on the gut microbial system in a pivotal time of microbial dynamics.

## Methods

### Study population and sampling

This prospective observational cohort included 61 children who first started attending day care for their first time in the 2018–2019 school year (September 2018 to July 2019). Four different day care facilities (day care A to D) were included, all are located within 10 kilometers from the lab. Stool samples and questioners were collected within the first 2 weeks after the child started consistent day care attendance (sample or time point 1), and after two, four, seven, and ten months (time points 2–5) during the course of a year (Fig. 1a). 24 additional age-matched children in home care settings were also included, those did not attended day care before or during the study sampling. Children in the four day care facilities live in the same geographic region in close proximity to the lab, and did not show significant differences in social economic status (tracked by the number of rooms in the household and parental education) and by model of delivery and breast feeding (Supplementary Table 2). All four day care facilities had internal home-made cooking, which served age-adjusted food with diverse ingredients including dairy products, fruits, vegetables, chicken, pasta, and rice. In Day care B and C there were 2 classes within each facility, with some of the caregivers working in both classes. We aimed to include children that entered day care for the first time, and were under 3 years old when entering day care. Parents and day care personal were instructed to keep and label diapers with stool content that were changed during the mornings of the specified collection days. Sampling of the stool at the lab was done instantly and samples were immediately frozen −80C until further analyses. All day care samples were handled and processed similarly and together and the home care samples were processed with the last day care sampling. We included negative controls (extraction and PCR blanks) that were prepared similarly and together with the rest of the samples. We excluded samples when subjects were on antibiotics within 6 weeks of stool collection. We have complied with all relevant ethical regulations for work with human participants. Ethical approval for the study was granted by the Sheba Research Ethics Committee. Written, informed consent was obtained.

### 16S rRNA gene amplicon sequencing and analyses

DNA extraction and PCR amplification of the variable region 4 (V4) of the 16S rRNA gene using Illumina adapted universal primers 515 F/806 R was conducted using the direct PCR protocol [Extract-N-Amp Plant PCR kit (Sigma-Aldrich, Inc.)]^36–38^. PCRs were conducted and amplicons were pooled in equimolar concentrations into a composite sample that was size selected (300–500 bp) using agarose gel to reduce non-specific products from host DNA. Sequencing was performed on the Illumina MiSeq platform with the addition of 15% PhiX, and generating paired-end reads of 175b in length in each direction.

Reads were processed in a data curation pipeline implemented in QIIME 2 version 2019.4^39,40^. Reads were demultiplexed according to sample specific barcodes. Quality control was performed by truncating reads after three consecutive Phred scores lower than 20. Reads with ambiguous base calls or shorter than 150 bp after quality truncation were discarded. Amplicon sequence variants (ASVs) detection was performed using Deblur^41^ and duplicate samples from different runs were joined, resulting in 268 samples with median of 20 K reads/sampe (IQR 13–27 K). ASV taxonomic classification was assigned using a naive Bayes fitted classifier, trained on the August 2013 Greengenes database, and on SILVA release 138 database^42^ for 99% identity 150 bp long reads^43^. Unweighted UniFrac was used as a measure of between sample β-diversity^44^, using a phylogenetic tree generated by SATé-enabled phylogenetic placement (SEPP)^45^. All samples were rarefied to 4000 reads for α and β diversity analysis, to avoid read number effects. The resulting distance matrix was used to perform a principal coordinates analysis (PCoA). heatmaps were generated using Calour version 2018.10.1 with default parameters^18^.

PERMANOVA: Quantifications of variance were calculated using PERMANOVA with the adonis2 function in the R package Vegan (vegan: Community Ecology Package. R package version 2.5–6. https://CRAN.R-project.org/package=vegan)^46^, on the rarefied Unweighted UniFrac distance values. The total variance explained by each variable was calculated while accounting for age and gender in the model (except for when looking at the contribution of age and gender, when only age or gender can be controlled for), and for subject using adonis strata in the longitudinal analysis. The per time point analysis included only one sample per patient, and therefore accounted only for age and gender.

Random forest analysis of home care and day care samples: ASVs of 24 home care and 24 manually matched day care samples were used, to avoid group size bias. The matching of day care samples was performed while accounting for age (up to 1 month of difference in age), mode of delivery, breastfeeding and the number of persons in the household (Supplementary Table 1). In addition, to further ensure that our results are not the results of a bias, we chose 100 random subsets of 24 daycare children out of the 60 available age-matched samples, and used them in a random forest analysis compared to our 24 home care children. The random forest analysis was performed in R package randomForest^47^. This class probability was used to calculate the AUC^48^. Score threshold was calculated using Youden index.

### Statistical analyses

Age correlated ASVs: Using only samples from the first sampling (i.e. within 2 weeks of entering day care, and hence with only one sample per participant), we identified ASVs significantly positively or negatively correlated with age by using a permutation-based spearman rank correlation with dsFDR multiple hypothesis correction (FDR < 0.1)^49^ as implemented in Calour.

Effect of day care facility using age-matched pairs: For each sampling point (1 to 5), each day care child sample was matched to samples of other day care children that are aged up to 1 month apart, and including only one sample per child. These samples were further divided into “same” and “different” day care groups based on the day care facility the two children attended. Binary-Jaccard distance was then calculated for all pairs within each group using equation (1) below:

$$D_{i,j} = 1 - \frac{{\left| {B_i \cap B_j} \right|}}{{\left| {B_i \cup B_j} \right|}}$$

where *D*_*i, j*_ denotes the Binary-Jaccard distance, and *B*_*i*_, *B*_*j*_ denote the bacteria present in samples *i, j* respectively.

For each sampling period, significance of the difference between the “same” and “different” day care distance distributions was calculated using the non-parametric Mann–Whitney test (as implemented in scipy version 1.5).

Home care and day care associated ASVs: differentially abundant ASVs (between day care and home care) were identified using a paired feature-wise non-parametric rank mean test as implemented in Calour^18^ with dsFDR multiple hypothesis correction (FDR < 0.1)^49^. For each feature (bacteria), the relative abundance across all samples is first ranked. The mean of the ranks for the bacteria in each group is then calculated, and the *p*-value is calculated by comparing to random permutations of the group labels, that are performed only within samples of similar pairing field values. Finally, dsFDR multiple hypothesis correction is applied for the *p*-values resulting from all the features. To account for age, and to avoid multiple sampling from the same participant, we created a set of age-matched day care children samples for each of the home care sample, with an age difference up to one month, and using only a single sample from each day care participant (Supplementary Fig. 6). The process was as follows: starting with the pool of all day care children samples, in each iteration go over all home care children, and for each home care child select the latest time point for day care child’s sample with an age difference less than 1 month. Then remove all day care samples of that child whose sample was already selected. This repeats until no more matching day care sample are found. The result of this age-matching is, for each home care child, a set of day care samples with an age up to 1 month from the home care child were captured. This process also removed the dependence between same child samples. This resulted in 60 samples from children in day care, which were age matched to the 24 home care samples (median age of 12 and 12.4 months for day care and home care respectively, Supplementary Fig. 6). A paired test was then used with grouping on the home care participant for whom the matching was performed (i.e., each group contained a single home care sample and the corresponding age-matched day care samples).

Identification of age associated ASVs in additional studies: per-sample FASTA reads files were downloaded from the SRA for two additional studies (^20,21^) that included child microbiome samples sequenced using the V4 region (accession numbers PRJEB20773 and PRJNA290380 respectively). Sequences were processed using the same pipeline described for the current dataset. For PRJEB20773^20^ we identified age-associated ASVs by comparing study samples of adults (*n* = 40) and infants (*n* = 84, 6–12 months with mean age 8 month and median 7.2 months) not receiving antibiotics, using the non-parametric rank mean test as implemented in Calour with dsFDR multiple hypothesis correction (FDR < 0.1). The second study, PRJNA290380^21^, included samples of children aged 0–1200 days (4 years) spanning three countries (Estonia, Russia, and Finland). For each country, we identified age-associated ASVs by testing for ASVs significantly correlated or anti-correlated with age using the Spearman rank-correlation with dsFDR correction (FDR < 0.1) implemented in Calour.

Alpha-diversity analysis: All samples were rarefied to 4000 reads for α and β diversity analysis. Number of observed ASVs was used as the alpha-diversity metric for home care and day care comparison. Samples were stratified to 5-month age bins, and in cases where the age bin contained more than one sample from the same participant, these samples were joined to a single sample (using mean frequency). Non-parametric Mann–Whitney test was then used for comparison between the home care and day care groups at each age bin independently.

Multivariate Association with Linear Models to test for day care class-specific ASVs: Differentially abundant ASVs between day care classes were tested using MaAsLin2 (Multivariate Association with Linear Models) R package version 1.0.0 (https://huttenhower.sph.harvard.edu/maaslin/) with an FDR of 0.05, controlling for age and gender, and for patient ID as a random effect^50^.

### Reporting summary

Further information on research design is available in the Nature Research Reporting Summary linked to this article.

## Supplementary information

SUPPLEMENTAL MATERIAL

Data source

REPORTING SUMMARY

## Data Availability

The study datasets were deposited at the National Center for Biotechnology Information as BioProject PRJNA660912.

## Code availability

Python analysis scripts associated with this paper are available at: https://github.com/amnona/paper-daycare.
