# Supplementary material for: Gut microbiome development in early childhood is affected by day care attendance
Source: NPJ Biofilms Microbiomes. 2022 Jan 11;8:2. doi: 10.1038/s41522-021-00265-w (PMC8752763; doi:10.1038/s41522-021-00265-w)
Supplement: Supplementary file 1 — SUPPLEMENTAL MATERIAL [file 41522_2021_265_MOESM1_ESM.pdf]

Microbiome Development in Early Childhood is affected by Day Care Attendance

Amnon Amir^[1] PhD, Ortal Erez-Granat^[1] MD, Tzipi Braun^[1] MS, Katya Sosnovski[1] MS, Rotem Hadar[1] MS, Marina BenShoshan[1] MS, Sophia Heiman[1] MD, Haya Abbas-Egbariya[1] MS, Efrat Glick Saar[1] PhD, Gilat Efroni[1] PhD, Yael Haberman*[1, 2] MD PhD

^First equal contribution
[1] Sheba Medical Center, Tel-HaShomer, affiliated with the Tel-Aviv University, Israel.
Tel. (+972)3-530288.
[2] Cincinnati Children's Hospital Medical Center and the University of Cincinnati College of Medicine, Cincinnati, OH, USA.

*Corresponding Author:
Yael Haberman, MD, PhD
Pediatric Gastroenterology, Hepatology & Nutrition Unit
The Edmond and Lily Safra Children's Hospital, Sheba Medical Center, Tel Hashomer, Israel
Telephone number: 972-3-5302692
Yael.Haberman@sheba.health.gov.il

Supplemental Figures

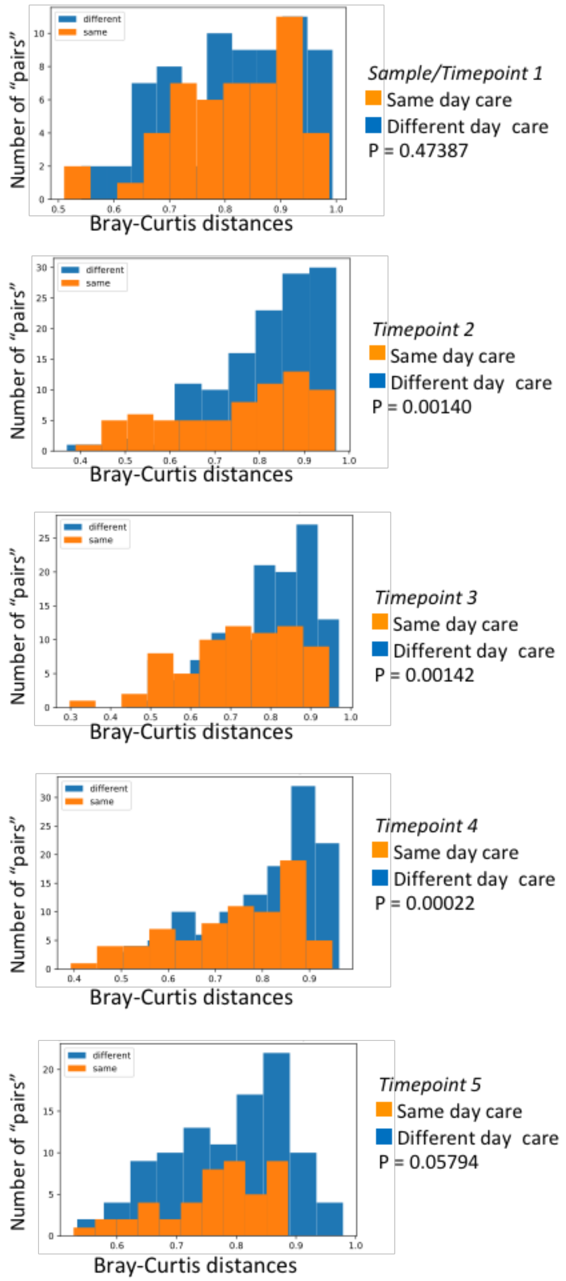

Supplementary Figure 1: Day care children become more similar to their classmates from the second sampling onward. Bray-Curtis distances (as opposed to Binary Jaccard distance in Figure 2A) was calculated between pairs of age-matched children (born within up to one month apart). Distances are shown for age-matched children pairs participating in the same day care (orange) or a different day care facility (blue), showing that from the second sampling onward, age-matched pairs from the same day care share more of their microbiome in comparison to pairs from different day care facilities. P-values for differences between same and different day care pairs were calculated using a two-sided Mann-Whitney test.

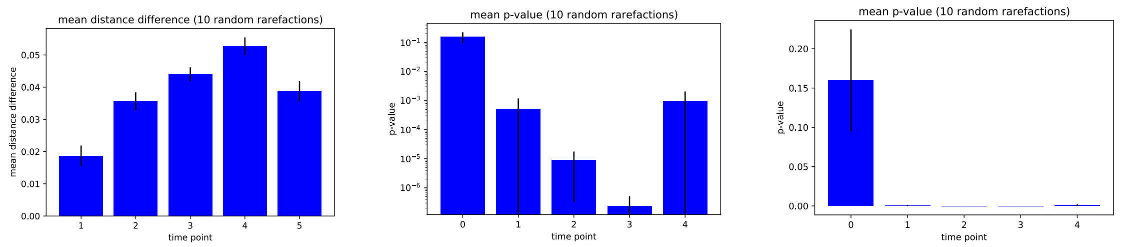

Supplementary Figure 2: Effect of multiple random rarefactions (4000 reads/sample) on the distances of age-matched children in similar vs. different day care facilities. Effect of sampling time point on the difference in the mean pair distances (left) between different and same day care facility child pairs, indicating different day care pairs are less similar than same day care pairs, in multiple random rarefactions. Corresponding p-value distributions (across 10 random rarefactions) for the null hypothesis of similar distance distribution for different and same day care facility pairs stratified for sampling time point in log (middle) and linear (right) scales. Bars represent standard diviations.

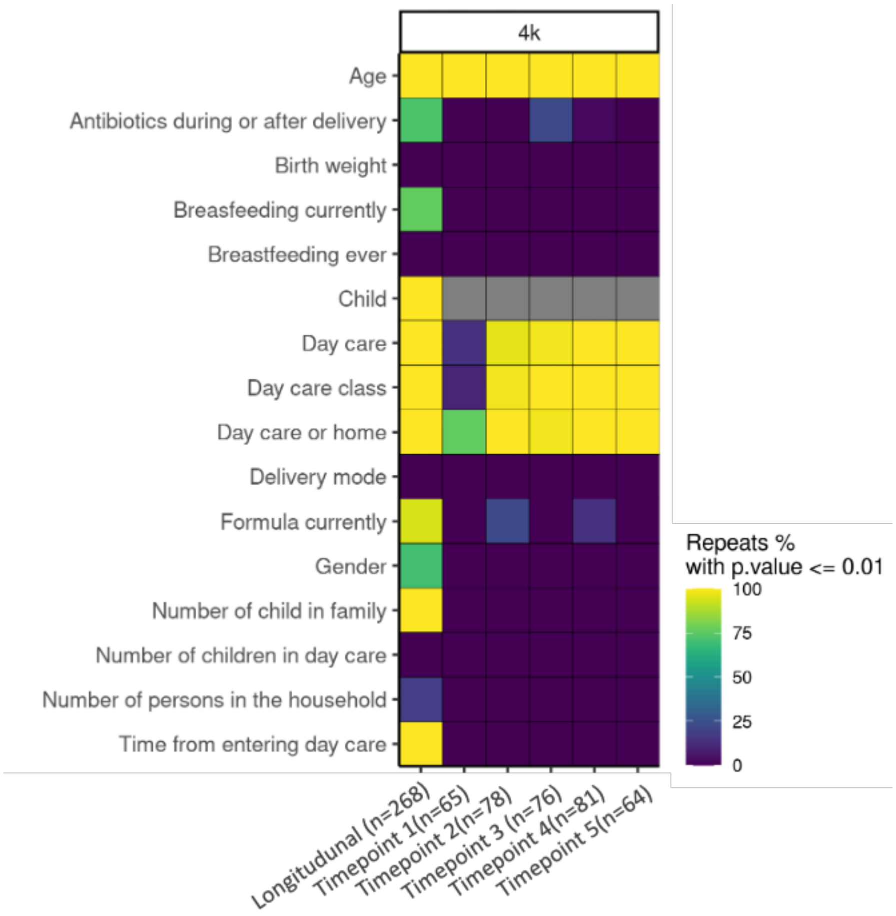

Supplementary Figure 3: Effect of multiple random rarefactions (4000 reads/sample) on thePermanova in Figure 2B. 50 random 4000 reads/sample rarefication with consistent results, and here we report in the heatmap the percent of repeats that showed p<0.01.

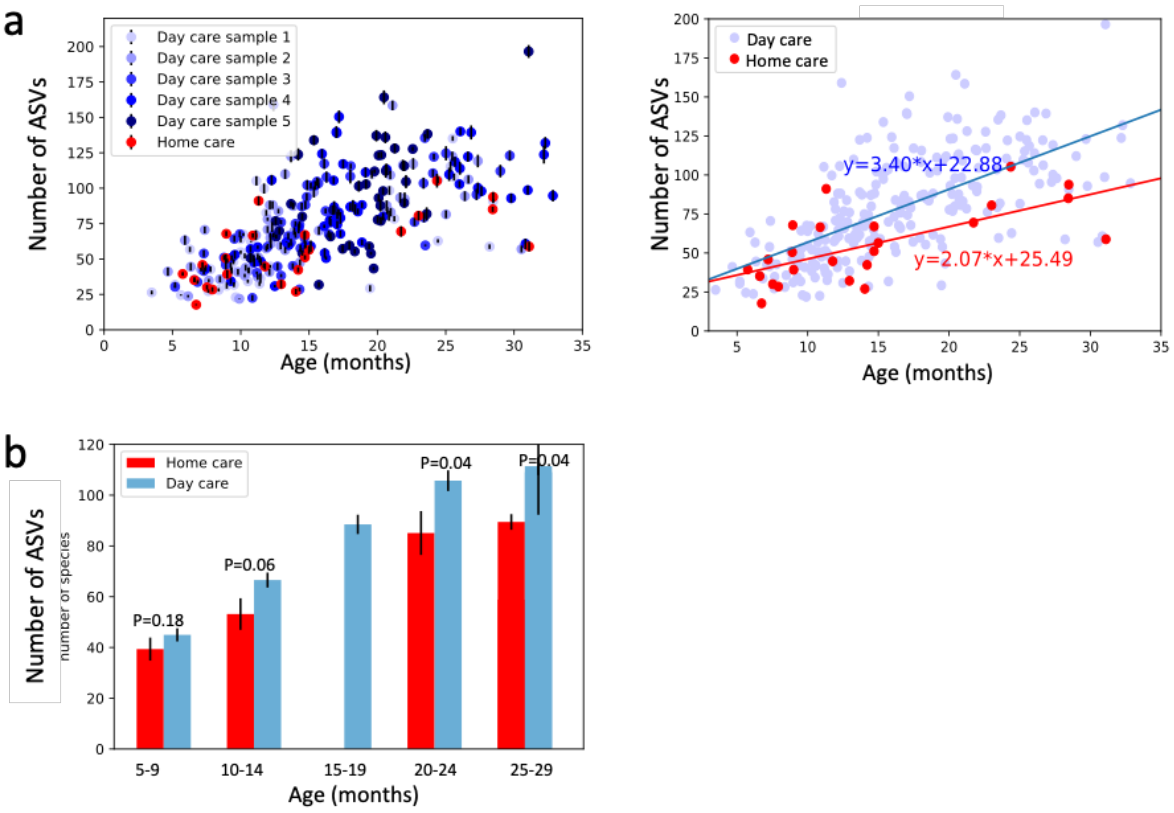

Supplementary Figure 4: Linear regression of alpha diversity versus age in day care and home care children. **Alpha diversity versus age in day care and home care children**. **a.** Number of amplicon sequence variants (ASVs) (average of 10 rarefactions to depth 4000 reads/sample) is plotted against age across day care (blue) and home samples (red) and indicating sampling number. Error bar within each circle denotes the standard deviation for the 10 rarefactions. Right panel: Linear regression was performed on the number of species (average of 10 rarefactions) as a function of age for the two groups with p-value=0.07 for the difference in the slopes by using a non-parametric single-sided test with 1000 random permutation of group labels (day care or home care). **b.** Bar plot indicating the mean and standard deviation stratified by age groups for home care (blue) and day care (red) children including samples from the second timepoint sampling onward. P-values above each age group indicate the day care vs. home care Mann-Whitney significance scores (based on the per-sample number of ASVs average over 10 rarefactions). Age 5-9months; 9 home care and 15 day care children, Age 10-14months; 9 home care and 41 daycare children, Age 20-24months; 3 home care and 26 daycare children, Age 25-29months; 3 home care and 18 day care children.

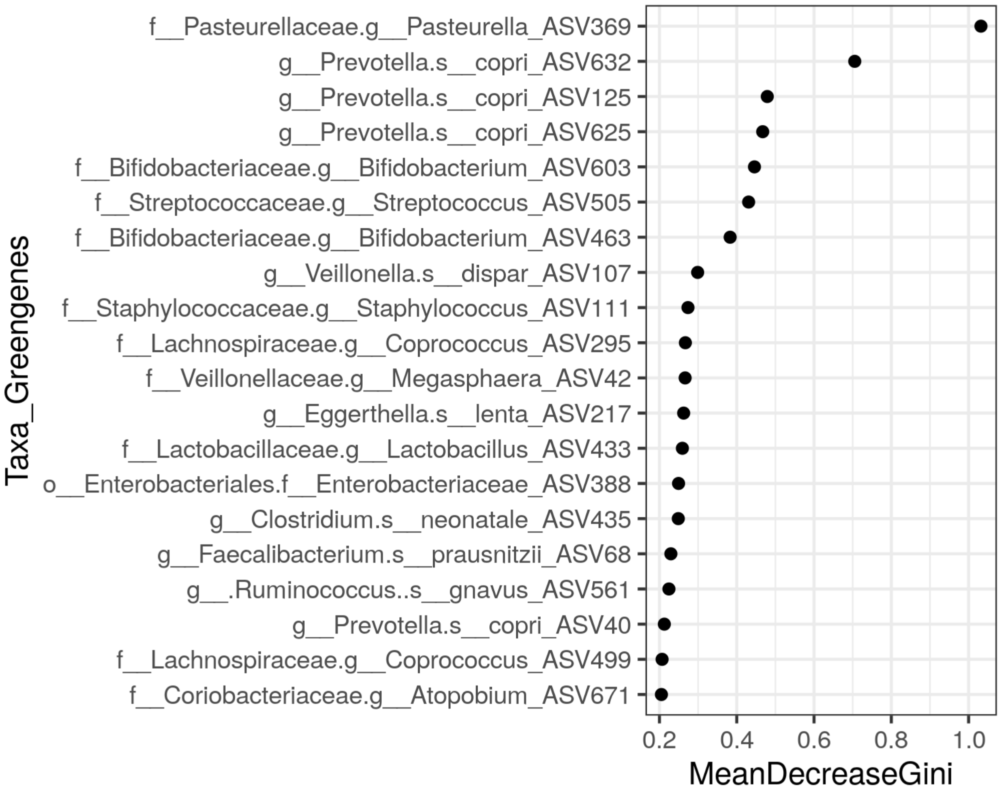

Supplementary Figure 5: Top 20 ASVs taxa used for the random forest classification in Figure 3A and B. Top 20 ASVs used for the random forest result differentiating between 24 home care and 24 age matched day care samples with an AUC of 0.88 as shown in Figure 3A and 3B.

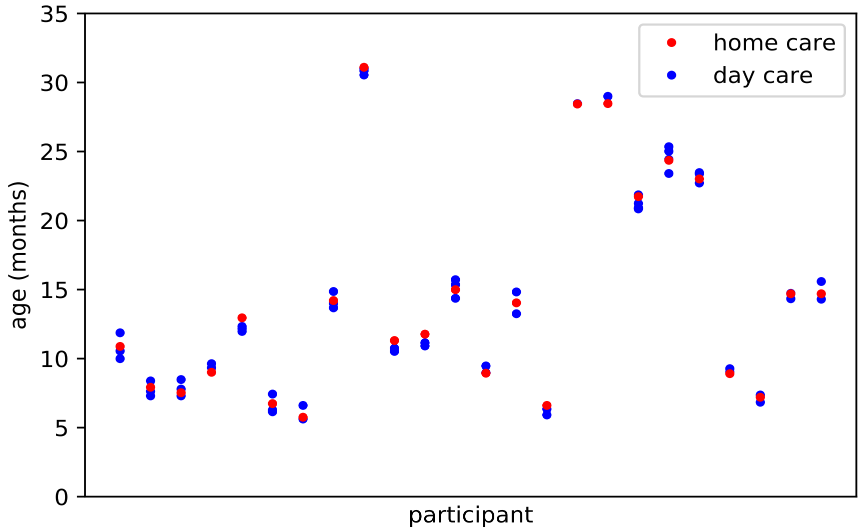

Supplementary Figure 6: Age matching of different home care and day care pairs used for the differential expression in Figure 3E-F. To account for age, and to avoid multiple sampling from the same participant we used aged-match pairs for analyses, whereby each home care child was matched with day care children that are up to 1 month apart in age, and only one sample per child was included (median age of 12 and 12.4 months for day care and home care matched groups respectively). Home care children in red and day care children in blue with their associated age as indicated

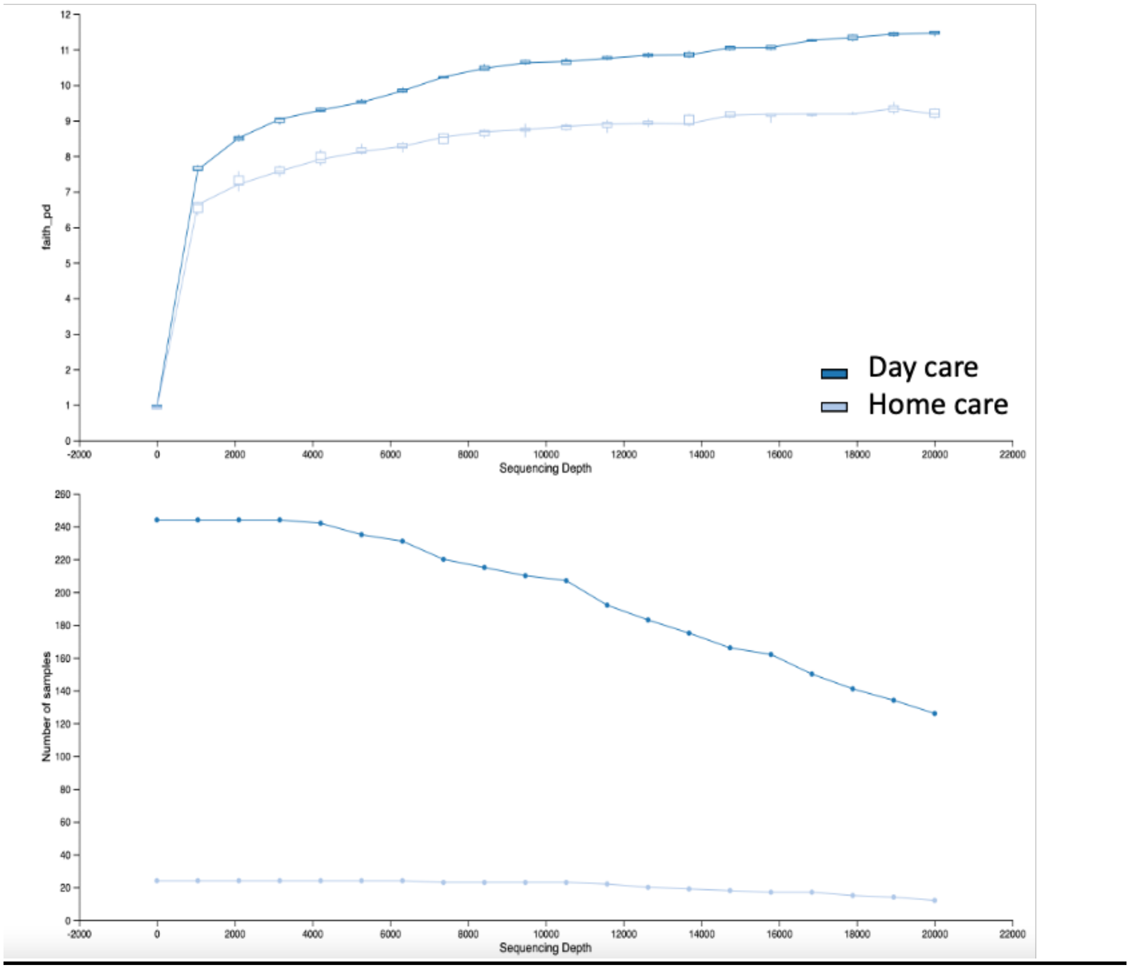

Supplementary Figure 7: Alpha rarefaction and read depth. Alpha rarefaction curves (upper panel) and a plot indicating the number of samples left after different rarefaction (lower panel).

Data Source (separate excel file) Description

Figure 1c: ASVs correlation with age using the first sample from each day care child
Figure 2b: PERMANOVA test to explain microbial variation
Figure 2c-d: Bacterial ASVs associated with day care class, as identified by Maaslin2
Figure 3a-b: Top 100 ASVs taxa used for the random forest classification
Figure 3c-d: Differentially abundant ASVs between age-matched day care and home care

Supplementary Tables

Supplementary Table 1: Demographics and characteristics for the 24 day care and 24 home care children used for the random forest result shown in Figure 3A and B.

	Day care (n=24)	Home care (n=24)
Age month Median (IQR)	11.4(8.9, 16.9)	12.4(8.7,16.7)
Male (n, %)	12(50%)	14(58%)
Persons in household	4(3,4)	4(3,4)
Never breast fed	11%	26%
Mode of delivery (vaginal)	92%	83%

Supplementary Table 2: Cohort demographics and characteristics by specific day care

Day care	A (n=11)	B (n=17)	C (n=22)	D (n=11)	Home (n=24)
Total number of children in day acre	11	27	40	14	NA
Male (%)	73%	47%	50%	36%	42%
Mode of delivery (vaginal)	82%	100%	81%	89%	83%
Never breast fed	11%	20%	0	22%	26%
Parental education (years, mean)	14.4	15.0	16.0	14.0	15.8
Maternal education (years, mean)	16.3	15.8	16.6	16.3	16.5
Rooms in the house (mean)	4.3	3.4	3.0	4.0	4.0
