# Supplementary material for: Gut microbiome development in early childhood is affected by day care attendance
Source: NPJ Biofilms Microbiomes. 2022 Jan 11;8:2. doi: 10.1038/s41522-021-00265-w (PMC8752763; doi:10.1038/s41522-021-00265-w)
Supplement: Supplementary file 3 — REPORTING SUMMARY [file 41522_2021_265_MOESM3_ESM.pdf]

nature portfolio

Corresponding author(s): Yael Haberman

Last updated by author(s): Nov 4, 2021

Reporting Summary

Nature Portfolio wishes to improve the reproducibility of the work that we publish. This form provides structure for consistency and transparency in reporting. For further information on Nature Portfolio policies, see our Editorial Policies and the Editorial Policy Checklist.

Statistics

For all statistical analyses, confirm that the following items are present in the figure legend, table legend, main text, or Methods section.

n/a	Confirmed	
☐	☒	The exact sample size (n) for each experimental group/condition, given as a discrete number and unit of measurement
☐	☒	A statement on whether measurements were taken from distinct samples or whether the same sample was measured repeatedly
☐	☒	The statistical test(s) used AND whether they are one- or two-sided<br>*Only common tests should be described solely by name; describe more complex techniques in the Methods section.*
☐	☒	A description of all covariates tested
☐	☒	A description of any assumptions or corrections, such as tests of normality and adjustment for multiple comparisons
☐	☒	A full description of the statistical parameters including central tendency (e.g. means) or other basic estimates (e.g. regression coefficient) AND variation (e.g. standard deviation) or associated estimates of uncertainty (e.g. confidence intervals)
☐	☒	For null hypothesis testing, the test statistic (e.g. F, t, r) with confidence intervals, effect sizes, degrees of freedom and P value noted<br>*Give P values as exact values whenever suitable.*
☒	☐	For Bayesian analysis, information on the choice of priors and Markov chain Monte Carlo settings
☒	☐	For hierarchical and complex designs, identification of the appropriate level for tests and full reporting of outcomes
☐	☒	Estimates of effect sizes (e.g. Cohen's d, Pearson's r), indicating how they were calculated

Our web collection on statistics for biologists contains articles on many of the points above.

Software and code

Policy information about availability of computer code

Data collection	PRJEB20773 and PRJNA290380
Data analysis	QIIME 2 ver 2019.4, Deblur, 2013 Greengenes database, SILVA release 138 database, SATé-enabled phylogenetic placement (SEPP), R package Vegan (vegan: Community Ecology Package), MaAsLin2, R package randomForest

For manuscripts utilizing custom algorithms or software that are central to the research but not yet described in published literature, software must be made available to editors and reviewers. We strongly encourage code deposition in a community repository (e.g. GitHub). See the Nature Portfolio guidelines for submitting code & software for further information.

Data

Policy information about availability of data

All manuscripts must include a data availability statement. This statement should provide the following information, where applicable:

- Accession codes, unique identifiers, or web links for publicly available datasets
- A description of any restrictions on data availability
- For clinical datasets or third party data, please ensure that the statement adheres to our policy

The study datasets were deposited at the National Center for Biotechnology Information as BioProject PRJNA660912

Field-specific reporting

Please select the one below that is the best fit for your research. If you are not sure, read the appropriate sections before making your selection.

☒ Life sciences ☐ Behavioural & social sciences ☐ Ecological, evolutionary & environmental sciences

For a reference copy of the document with all sections, see nature.com/documents/nr-reporting-summary-flat.pdf

Life sciences study design

All studies must disclose on these points even when the disclosure is negative.

Sample size	We aimed to include at least 50 day care and 20 home care children. We were unable to preform power calculation due to lack of other studies with similar designs (longitudunal day care).
Data exclusions	Exclusion criteria for samples included sample with under 4000reads/saple. We di dnot include samples form subjects treated with antibiotic within 6 weeks of sampling.
Replication	Finding were not replicated due to lack of similar day care study design
Randomization	All day care samples were handled and processed similarly and together and the home care samples were processed with the last day care sampling.
Blinding	Blinding was not relevant

Reporting for specific materials, systems and methods

We require information from authors about some types of materials, experimental systems and methods used in many studies. Here, indicate whether each material, system or method listed is relevant to your study. If you are not sure if a list item applies to your research, read the appropriate section before selecting a response.

Materials & experimental systems

n/a	Involved in the study	
☒	☐	Antibodies
☒	☐	Eukaryotic cell lines
☒	☐	Palaeontology and archaeology
☒	☐	Animals and other organisms
☐	☒	Human research participants
☒	☐	Clinical data
☒	☐	Dual use research of concern

Methods

n/a	Involved in the study	
☒	☐	ChIP-seq
☒	☐	Flow cytometry
☒	☐	MRI-based neuroimaging

Human research participants

Policy information about studies involving human research participants

Population characteristics	This is a prospective observational cohort study of 61 children (Male and female) who entered day care in the school year 2018-2019 (September 2018 to July 2019) in 4 different day cares. 24 additional age-matched children in home care setting were also included. All children were 6month to 4years old.
Recruitment	Four different day care facilities were included, all are located non-rural area in the central part of Israel within 10 kilometers from the lab at the Sheba Medical Center.
Ethics oversight	Ethical approval for the study was granted by the Sheba Research Ethics Committee

Note that full information on the approval of the study protocol must also be provided in the manuscript.
